# PCBP2 inhibits antiviral innate immune responses via the MAVS-mediated signaling pathway in severe fever with thrombocytopenia syndrome

**DOI:** 10.1016/j.virusres.2026.199699

**Published:** 2026-02-02

**Authors:** Xinyi Yu, Yan Dai, Xuewen Ji, Qinqin Pu, Ruonan Zhang, Mengqi Shi, Nannan Hu, Ke Jin, Jin Zhu, Jun Li

**Affiliations:** a Department of Infectious Disease, The First Affiliated Hospital with Nanjing Medical University, Nanjing, China; b School of Medicine and Holistic Integrative Medicine, Nanjing University of Chinese Medicine, Nanjing, China; c The First Affiliated Hospital With Nanjing Medical University, Nanjing, China; d Huadong Medical Institute of Biotechniques, Nanjing, China

**Keywords:** SFTS, Dabie bandavirus, PCBP2, Innate immunity, RIG-I-like receptor signaling pathway, MAVS

## Abstract

**Background:**

Severe fever with thrombocytopenia syndrome (SFTS) is an emerging tick-borne infectious disease with high mortality and limited therapeutic options. Poly(rC)-binding protein 2 (PCBP2) is a multifunctional RNA-binding protein involved in post-transcriptional regulation and innate immune modulation. Although PCBP2 has been reported to negatively regulate antiviral signaling in other viral infections, its role in Dabie bandavirus (DBV) infection remains unclear.

**Methods:**

Single-cell transcriptomic analysis was performed to characterize PCBP2 mRNA expression across immune cell populations in peripheral blood. PCBP2 expression levels were further examined in DBV-infected clinical samples, IFNAR⁻/⁻ mouse models, and THP-1 cells using quantitative RT-PCR, western blotting, and immunohistochemistry. THP-1 cells with plasmid-mediated PCBP2 overexpression or lentivirus-mediated PCBP2 knockdown were established to investigate the functional role of PCBP2. Activation of the RIG-I-like receptor (RLR) signaling pathway was evaluated by assessing key signaling molecules and downstream interferon responses. The impact of PCBP2 on DBV replication was determined by TCID_50_ assay, viral nucleoprotein (NP) expression, and immunofluorescence analysis.

**Results:**

PCBP2 expression was significantly downregulated during DBV infection in clinical samples, animal models, and cell cultures, and reduced PCBP2 expression was associated with increased disease severity and unfavorable clinical outcomes. Functional analyses demonstrated that PCBP2 suppressed DBV-induced activation of type I interferon signaling and interferon-stimulated genes, including ISG12a and G1P3. Mechanistically, PCBP2 directly interacted with the mitochondrial antiviral signaling protein MAVS and promoted its K48-linked polyubiquitination, resulting in proteasome-dependent degradation and attenuation of the MAVS-TBK1-IRF3 signaling axis. Consistent with its immunosuppressive role, PCBP2 knockdown significantly reduced DBV replication, whereas PCBP2 overexpression enhanced viral replication in THP-1 cells.

**Conclusions:**

These findings identify PCBP2 as a critical negative regulator of RLR-mediated antiviral signaling during DBV infection. By facilitating MAVS degradation and suppressing innate immune responses, PCBP2 promotes viral replication, providing new insights into DBV immune evasion mechanisms and highlighting PCBP2 as a potential host-directed therapeutic target for SFTS.

## Highlights

• PCBP2 is downregulated in SFTS and negatively correlates with disease severity.
• PCBP2 suppresses DBV-triggered IFN-β and ISG production in human monocytes.
• PCBP2 directly binds MAVS and induces its K48-linked polyubiquitination.
• PCBP2 promotes DBV replication by degrading MAVS and inhibiting innate immunity.
• PCBP2 is a potential therapeutic target for controlling SFTS virus infection.

**Importance**

Severe fever with thrombocytopenia syndrome (SFTS), caused by Dabie bandavirus (DBV), is an emerging tick-borne infectious disease associated with high mortality and poses a serious public health threat in East Asia. The absence of approved vaccines or specific antiviral therapies highlights the urgent need to better understand host–virus interactions underlying DBV pathogenesis. Poly(rC)-binding protein 2 (PCBP2) is a multifunctional RNA-binding protein involved in post-transcriptional regulation and innate immune modulation. However, its role in DBV infection has not been fully elucidated. In this study, we demonstrate that PCBP2 acts as a negative regulator of DBV-triggered innate immune responses. PCBP2 directly interacts with the mitochondrial antiviral signaling protein MAVS and promotes its K48-linked polyubiquitination and proteasomal degradation, thereby suppressing type I interferon signaling and facilitating DBV replication. These findings identify PCBP2 as a critical host factor regulating antiviral immunity during DBV infection and reveal a previously unrecognized immune evasion strategy employed by bunyaviruses.

Alt-text: Unlabelled box dummy alt text

## 1 Introduction

Severe fever with thrombocytopenia syndrome (SFTS) is an emerging tick-borne infectious disease caused by Dabie bandavirus (DBV), posing a serious threat to public health in East Asia (Gai et al., 2012). Clinically, SFTS presents with a broad disease spectrum, ranging from self-limiting febrile illness with thrombocytopenia and leukocytopenia to severe complications such as disseminated intravascular coagulation and multiple organ failure (Lu et al., 2022). Since its initial identification in China, SFTS cases have been increasingly reported in South Korea and Japan, with a case fatality rate ranging from 6% to 30% (Kim et al., 2013). The high morbidity and mortality associated with SFTS, together with its expanding geographic distribution, have made it an urgent public health concern (Li et al., 2018).

Accumulating clinical evidence indicates that viral load is a critical determinant of disease severity and clinical outcome in SFTS patients. Individuals with high DBV viral loads exhibit significantly increased mortality rates compared with those harboring lower viral burdens. Although a randomized double-blind clinical trial in China demonstrated that favipiravir treatment markedly reduced mortality in patients with low viral loads, no significant benefit was observed in patients with high viral loads (Li et al., 2021). These findings underscore the urgent need to elucidate the mechanisms by which DBV evades host antiviral immunity and establishes efficient replication, particularly in severe disease settings.

Poly(rC)-binding protein 2 (PCBP2) is a multifunctional cellular RNA-binding protein involved in post-transcriptional regulation, including mRNA stabilization and translational control (Ren et al., 2016). In addition to its role in RNA metabolism, PCBP2 acts as a cytosolic iron chaperone that coordinates intracellular iron trafficking, thereby influencing multiple cellular processes (Philpott et al., 2020). Aberrant expression of PCBP2 has also been implicated in tumorigenesis and malignant progression in several cancers (Chen et al., 2018). In the context of viral infection, PCBP2 has been reported to participate in viral life cycles by regulating the transition from viral translation to RNA replication (Perera et al., 2007).

Importantly, previous biochemical and immunological studies have identified PCBP2 as a negative regulator of mitochondrial antiviral signaling protein (MAVS)-mediated innate immune responses (You et al., 2009). By targeting key components of the RIG-I-like receptor (RLR) signaling pathway, PCBP2 restrains excessive type I interferon activation and maintains immune homeostasis (Qin et al., 2017). While such immunoregulatory functions may protect the host from immune-mediated damage, they may also be exploited by viruses to facilitate immune evasion and enhance viral replication.

Despite these advances, the role of PCBP2 in DBV infection and SFTS pathogenesis remains largely unexplored. In particular, it is unclear whether alterations in PCBP2 expression occur during DBV infection and how such changes are associated with viral replication and disease severity. In this study, we employed both in vivo and in vitro DBV infection models to systematically investigate the expression dynamics and functional role of PCBP2 during infection. Our findings reveal that PCBP2 is downregulated in DBV-infected hosts and cells, and that reduced PCBP2 expression is associated with enhanced disease severity. Mechanistically, we demonstrate that PCBP2 modulates DBV replication by regulating MAVS-mediated innate immune signaling. These insights provide a mechanistic framework for understanding DBV immune evasion and highlight PCBP2 as a potential host-targeted therapeutic candidate for SFTS.

## 2 Materials and methods

### 2.1 Study population

Thirty moderate and thirty severe SFTS patients were enrolled from the Department of Infectious Diseases, First Affiliated Hospital of Nanjing Medical University between January 2023 and December 2024.

The inclusion criteria and clinical classification of SFTS patients were based on the Diagnosis and Treatment Consensus of Severe fever with thrombocytopenia syndrome (Infectious Diseases Society of Chinese Medical Association, 2022). Patients were diagnosed with SFTS by a Nucleic Acid Quantitative Assay Kit (DAAN GENE, Guangzhou, China) were selected for the study. Those subjects who had co-infection with HCV, HBV or HIV infection, tumors, and autoimmune diseases were excluded. According to the median of the expression of PCBP2, they were divided into low expression group and high expression group. Thirty healthy volunteers were also recruited into the study to serve as controls from the health promotion center of the First Affiliated Hospital of Nanjing Medical University, with confirmed absence of acute or chronic diseases upon medical evaluation. Written informed consent was obtained from all patients/participants.

### 2.2 Ethics statement

This study complied with the ethical guidelines of the Declaration of Helsinki, and was approved by the Ethics Committee of the First Affiliated Hospital of Nanjing Medical University (Protocol No. 2023-SR-336). All animal-related experiments were conducted in animal biological safety level 2+ (ABSL-2+) containment laboratories in Huadong Medical Institute of Biotechniques (HMIB). The mouse experiments were approved by the HMIB ethics committee and conducted in accordance with HMIB's ethical regulations for mouse experiments.

### 2.3 Isolation of human PBMCs

The protocol for isolating PBMCs was as follows. In brief, whole blood from SFTS patients was diluted in phosphate-buffered saline (PBS) (Sangon Biotech, Shanghai, China), and PBMC were isolated by centrifugation on a Ficoll-Hypaque density gradient (TBD Sciences, Tianjin, China). The cell layer of the PBMCs was transferred to a new centrifuge tube, and the cells were further diluted with PBS. Red blood cells were removed from the samples via red blood cell lysis (Sigma-Aldrich, Missouri**,** USA). Purified PBMCs were washed three times with PBS and pelleted at 800 × g for 5 min.

### 2.4 Viruses and cells

The DBV strain JS14 was provided by the Jiangsu Provincial Center for Disease Control and Prevention.

THP-1 and Vero cells were obtained from the Shanghai Cell Bank of the Chinese Academy of Sciences (Shanghai, China). THP-1 cells were cultured in 1640 medium (Gibco, New York, USA) supplemented with 10% FBS (Corning, New York, USA) and 1% penicillin-streptomycin (Gibco, New York, USA), while Vero cells were cultured in DMEM (Gibco, New York, USA). These cell were incubated in an incubator with 5% CO_2_ at 37°C.

All DBV were propagated in Vero cells and stored at -80°C until use.

### 2.5 Quantification of DBV viral load by quantitative RT-PCR (expressed as TCID_50_ equivalents)

DBV viral load was quantified using a commercial DBV nucleic acid quantitative assay kit (DAAN GENE, Guangzhou, China) according to the manufacturer’s instructions. This assay is based on quantitative RT-PCR and reports viral RNA levels as TCID_50_ equivalents per milliliter using a standardized calibration curve provided with the kit. The reported TCID_50_ values therefore represent RNA-based equivalent viral titers rather than infectious virus titers determined by cell culture.

Briefly, quantitative standards supplied with the kit were used to generate a standard curve. Samples were subjected to viral lysis, and the lysates were transferred to nucleic acid binding columns. After centrifugation at 12,000 × g for 1 min, viral RNA was allowed to bind specifically to the silica membrane. Impurities were removed by washing with 500 μL of wash buffer, and high-purity viral RNA was subsequently eluted using 35 μL of preheated elution buffer.

Quantitative RT-PCR was performed on an ABI 7500 real-time PCR system under the following cycling conditions: 50°C for 15 min, 95°C for 15 min, followed by 45 cycles of 94°C for 15 s and 55°C for 45 s, with fluorescence signals collected during each cycle. Viral load was calculated based on the standard curve and expressed as TCID_50_ equivalents.

### 2.6 Animal experiments

IFNAR⁻/⁻ mice were used in this study because wild-type mice are largely resistant to DBV infection, whereas deficiency of type I interferon signaling permits robust viral replication and reproducible disease manifestations in vivo. This model has therefore been widely employed to investigate DBV pathogenesis and host–virus interactions.

IFNAR^−/−^ mice were purchased from Cyagen Biosciences Inc. (Suzhou, China) and maintained under specific-pathogen-free (SPF) conditions in an environmentally controlled animal facility at Huadong Medical Institute of Biotechniques (HMIB). Six- to eight-week-old mice were randomly assigned into three groups (n = 3 per group): a mock-infected control group, a low-dose DBV infection group, and a high-dose DBV infection group.

On day 0, mice in the low-dose and high-dose groups were subcutaneously inoculated with 100 μL of DBV at doses of 1.0 × 10^4^ TCID_50_ equivalents/mL and 1.0 × 10⁶ TCID_50_ equivalents/mL, respectively. Mock-infected mice received an equal volume of DMEM via the same route.

Body weight was measured and recorded daily for each mouse as an indicator of disease progression. Peripheral blood samples were collected via the orbital venous plexus on days 1, 3, 5, and 7 post-infection for the measurement of viral RNA load and PCBP2 mRNA expression by quantitative RT-PCR. On day 7 post-infection, all mice were euthanized, and the liver, spleen, kidney, and intestine were harvested for immunohistochemical analysis.

### 2.7 Western blot and immunoprecipitation (IP) assays

Total protein from cultured cells or mouse tissues was extracted using RIPA lysis buffer supplemented with protease and phosphatase inhibitor cocktails (Beyotime, Shanghai, China) and quantified using a bicinchoninic acid (BCA) protein assay kit according to the manufacturer’s instructions. Equal amounts of protein were denatured at 100°C for 10 min, separated by 12% SDS–polyacrylamide gel electrophoresis (SDS-PAGE), and transferred onto polyvinylidene fluoride (PVDF) membranes (Millipore, MA, USA).

After blocking with 5% non-fat milk for 1 h at room temperature, membranes were incubated with primary antibodies at 4°C overnight, followed by incubation with HRP-conjugated secondary antibodies for 1 h at room temperature. Protein bands were visualized using enhanced chemiluminescence (ECL) reagents (Tanon, Shanghai, China). Band intensities were quantified by densitometric analysis using ImageJ software and normalized to β-actin. All western blot experiments were performed independently at least three times.

For co-immunoprecipitation assays, transfected cells were lysed in non-denaturing lysis buffer containing protease and phosphatase inhibitors. Cell lysates were clarified by centrifugation, and equal amounts of supernatants were incubated with anti-PCBP2 antibody or control IgG overnight at 4°C, followed by incubation with Protein A/G PLUS-Agarose beads. After extensive washing with TBS, the immunoprecipitated proteins were eluted and subjected to western blot analysis. Input lysates were analyzed in parallel to confirm protein expression levels.

### 2.8 RNA extraction and qRT-PCR

Total RNA was extracted from cultured cells, peripheral blood mononuclear cells (PBMCs), or mouse tissues using the RNAfast200 kit (Fastagen, Shanghai, China) according to the manufacturer’s instructions. First-strand cDNA was synthesized using PrimeScript RT Master Mix (Takara, Kyoto, Japan).

Quantitative real-time PCR was performed using TB Green Premix Ex Taq (Takara, Kyoto, Japan) on an ABI QuantStudio 5 Real-Time PCR System (Thermo Fisher Scientific, MA, USA). GAPDH was used as an endogenous control for normalization, and its expression was confirmed to be stable across different experimental conditions. Relative gene expression levels were calculated using the 2^⁻ΔΔCt^ method.

All qRT-PCR experiments were performed with at least three independent biological replicates, and each sample was analyzed in technical triplicate. Primer sequences were synthesized by Sangon Biotech (Shanghai, China) and are listed in Table S1.

### 2.9 Immunohistochemistry (IHC) analysis

Mouse tissues were fixed in 4% paraformaldehyde, embedded in paraffin, and sectioned at a thickness of 4 μm. Sections were deparaffinized, rehydrated, subjected to antigen retrieval, and blocked to reduce nonspecific binding. The sections were then incubated with a primary antibody against PCBP2 at 4°C overnight, followed by incubation with an HRP-conjugated secondary antibody for 30 min at room temperature.

Immunoreactivity was visualized using diaminobenzidine (DAB) substrate, and nuclei were counterstained with hematoxylin. Negative control sections were processed in parallel by replacing the primary antibody with isotype-matched IgG. Images were captured using a BioTek Cytation 5 imaging system (BioTek, VT, USA). Staining intensity and the percentage of PCBP2-positive cells were evaluated in a blinded manner to assess relative PCBP2 expression levels.

### 2.10 Immunofluorescence

THP-1 cells were infected with DBV and, as indicated, either transiently transfected with PCBP2 overexpression plasmids or established as PCBP2 knockdown cell lines via lentiviral transduction. At 24 h post-infection, cells were directly smeared onto glass slides, fixed with 4% paraformaldehyde for 10 min, and permeabilized with 0.02% Triton X-100 (Solarbio, Beijing, China). After washing with phosphate-buffered saline (PBS), cells were blocked with blocking buffer for 30 min at room temperature and then incubated overnight at 4°C with primary antibodies against PCBP2 and MAVS.

Following three washes with PBS, cells were incubated with species-specific fluorophore-conjugated secondary antibodies for 1 h at room temperature in the dark. Nuclei were counterstained with 4′,6-diamidino-2-phenylindole (DAPI) (Invitrogen, CA, USA). Fluorescence images were acquired using an Olympus IX73 inverted fluorescence microscope (Olympus, Tokyo, Japan). Images shown are representative of at least three independent experiments, and all images were captured using identical exposure settings.

### 2.11 Cell transfection and generation of stable cell lines

Plasmids encoding Flag-tagged PCBP2, HA-tagged ubiquitin, and HA-tagged K48-linked ubiquitin were commercially constructed by GenePharma (Shanghai, China). For PCBP2 overexpression, THP-1 cells were transiently transfected with Flag-PCBP2 plasmids (hereafter referred to as PCBP2^OE^) using Lipofectamine 2000 (Invitrogen, CA, USA) according to the manufacturer’s instructions. Cells were harvested 48 h post-transfection, and transfection efficiency was confirmed by quantitative RT-PCR and western blot analysis.

For the generation of stable PCBP2 knockdown cell lines, lentiviral vectors encoding short hairpin RNA targeting PCBP2 (shPCBP2) were constructed and packaged by GenePharma (Shanghai, China). THP-1 cells were infected with lentiviruses and subsequently cultured in RPMI 1640 medium. Stable cell lines were selected using puromycin (10 μg/mL; MCE, NJ, USA), and PCBP2 knockdown efficiency was validated by qRT-PCR and western blot analysis.

### 2.12 Antibodies

β-Actin (13E5) rabbit mAb (Cat# 4970, 1:1000), rabbit anti-phospho-IRF3 (Cat# 4947, 1:1000), rabbit anti-IRF3 (Cat# 4302, 1:1000), rabbit anti-phospho-TBK1 (Cat# 5483, 1:1000), HA-Tag (C29F4) Rabbit mAb (Cat#3724,1:1000) and rabbit anti-TBK1 (Cat# 3504, 1:1000) were obtained from Cell Signaling Technology. Rabbit anti-PCBP2 (Cat# 15070-1-AP, 1:1000), anti-MAVS polyclonal antibodies (Cat# 14341-1-AP, 1:1000) were obtained from Proteintech.

### 2.13 Chemicals

MG132 (HY-13259) was purchased from MCE. Dimethyl sulfoxide (DMSO) (D8371) was purchased from Solarbio.

### 2.14 Statistical analysis

All data are presented as the mean ± standard deviation (SD). Statistical analyses were performed using GraphPad Prism 9 software (GraphPad Software, CA, USA). Comparisons between two groups were conducted using an unpaired two-tailed Student’s t-test. For comparisons among multiple groups, one-way analysis of variance (ANOVA) followed by appropriate post hoc tests was applied. For experiments involving time-course measurements, statistical analyses were performed at individual time points.

Survival curves were analyzed using the Kaplan–Meier method, and differences between groups were assessed by the log-rank test. A P value < 0.05 was considered statistically significant. “ns” indicates no statistically significant difference.

## 3 Results

### 3.1 PCBP2 expression is downregulated in SFTS PBMCs and is associated with disease severity and clinical outcome

**Fig. 1:**
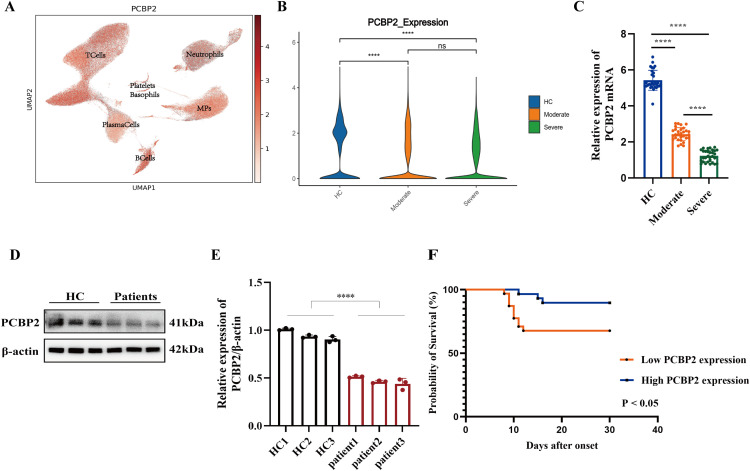
PCBP2 expression is downregulated in SFTS and is associated with disease severity and clinical outcome. (A) Uniform Manifold Approximation and Projection (UMAP) plot showing the distribution of PCBP2 mRNA expression across all single-cell clusters. (B) Single-cell transcriptomic analysis illustrating PCBP2 mRNA expression patterns in cells derived from healthy controls (HC), moderate SFTS patients, and severe SFTS patients. (C) Relative PCBP2 mRNA expression levels in peripheral blood mononuclear cells (PBMCs) from healthy controls and SFTS patients, as determined by qRT-PCR. (D) PCBP2 protein expression levels in PBMCs from healthy controls and SFTS patients, as detected by western blot analysis. (E) Densitometric quantification of PCBP2 protein levels in PBMCs shown in panel (D), normalized to β-actin and analyzed using ImageJ software. (F) Kaplan–Meier survival curves comparing SFTS patients with high and low PCBP2 expression.Data are presented as mean ± SD for applicable quantitative experiments. Statistical significance is indicated as follows: *, *P* < 0.05; **, *P* < 0.01; ***, *P* < 0.001; ****, *P* < 0.0001.

After batch-effect correction, single-cell transcriptomic data were subjected to unsupervised clustering analysis and visualized using Uniform Manifold Approximation and Projection (UMAP). The UMAP plot illustrates the distribution of PCBP2 mRNA expression across different cellular clusters (Fig. 1A). In addition, the expression patterns of PCBP2 at the single-cell transcriptomic level in cells derived from healthy controls, moderate SFTS patients, and severe SFTS patients are shown in Fig. 1B.

To further validate the single-cell transcriptomic findings at the bulk level, PCBP2 mRNA expression in peripheral blood mononuclear cells (PBMCs) was examined by qRT-PCR. Consistent with the single-cell results, PCBP2 mRNA levels were significantly reduced in SFTS patients compared with healthy controls, with lower expression observed in severe cases (Fig. 1C). Western blot analysis was subsequently performed to assess PCBP2 protein expression and confirmed an overall decrease in PCBP2 protein levels in SFTS patients relative to healthy controls (Fig. 1D). As no statistically significant difference in PCBP2 protein expression was observed between moderate and severe patients, these two groups were combined for comparison with the healthy control group in the western blot analysis. Densitometric quantification of the immunoblot signals further validated this reduction, showing a significant decrease in PCBP2 protein levels in SFTS patients after normalization to β-actin (Fig. 1E).

To explore the clinical relevance of PCBP2 expression, patients were stratified into high- and low-expression groups according to the median PCBP2 expression value (cut-off = 1.75). Kaplan–Meier survival analysis revealed that lower PCBP2 expression was significantly associated with unfavorable clinical outcomes in SFTS patients (Fig. 1F).

### 3.2 Expression of PCBP2 decreases during DBV infection in vivo and in vitro

Using this established susceptible mouse model, we examined the regulation of PCBP2 expression during DBV infection in vivo. IFNAR⁻/⁻ mice were randomly divided into three groups and subcutaneously inoculated with DBV at a low dose (10^4^ TCID_50_/mL), a high dose (10^6^ TCID_50_/mL), or with DMEM as a mock control. Peripheral blood samples were collected at 1, 3, 5, and 7 days post-infection (dpi) to determine viral load and PCBP2 mRNA expression levels.

**Fig. 2:**
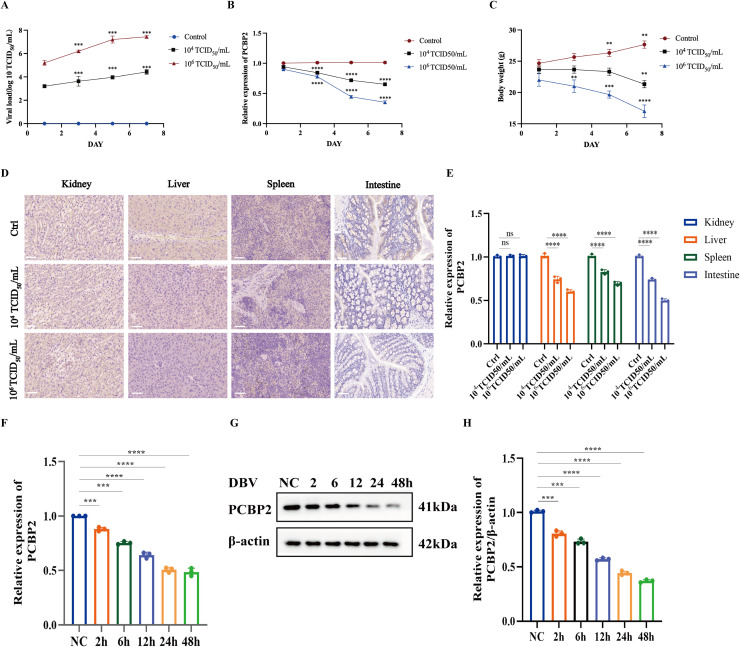
PCBP2 expression is progressively downregulated during DBV infection in vivo and in vitro. (A) Viral load in peripheral blood of IFNAR⁻/⁻ mice infected with DBV at low (10^4^ TCID_50_/mL) or high (10^6^ TCID_50_/mL) doses, measured at 1, 3, 5, and 7 days post-infection. (B) PCBP2 mRNA expression levels in peripheral blood of DBV-infected mice at the indicated time points. (C) Changes in body weight of mice following DBV infection. (D) Representative immunohistochemical staining of PCBP2 in liver, spleen, kidney, and intestine tissues collected at 7 dpi. (E) Quantification of PCBP2-positive staining in tissue sections. (F) PCBP2 mRNA levels in THP-1 cells infected with DBV (MOI = 1) at the indicated time points. (G) Western blot analysis of PCBP2 protein expression in DBV-infected THP-1 cells. (H) Densitometric quantification of PCBP2 protein levels normalized to β-actin. Data are presented as mean ± SD. *, *P* < 0.05; **, *P* < 0.01; ***, *P* < 0.001; ****, *P* < 0.0001. NC, negative control. ns, not significant.

As shown in Fig. 2A, DBV viral loads in peripheral blood increased progressively over time in infected mice, with consistently higher levels observed in the high-dose group compared with the low-dose group. In parallel, PCBP2 mRNA expression exhibited a gradual decline during DBV infection, which was more pronounced in mice receiving the higher viral dose (Fig. 2B). Body weight monitoring further demonstrated dose-dependent disease progression, with mice in the high-dose group displaying more evident weight loss over time (Fig. 2C).

At 7 dpi, mice were euthanized and major target organs, including liver, spleen, kidney, and intestine, were harvested for immunohistochemical analysis. PCBP2-positive staining was markedly reduced in DBV-infected mice compared with mock controls, particularly in the liver and spleen, and this reduction was more evident in the high-dose group (Fig. 2D). Quantitative analysis of IHC staining confirmed a significant decrease in PCBP2 expression in these organs following DBV infection (Fig. 2E).

To validate these observations in vitro, THP-1 cells were infected with DBV at a multiplicity of infection (MOI) of 1 and harvested at 2, 6, 12, 24, and 48 hours post-infection. qRT-PCR and western blot analyses revealed that PCBP2 mRNA and protein levels remained relatively stable at early time points (2 and 6 h), began to decline at 12 h, and were markedly reduced at 24 h and 48 h post-infection (Fig. 2F–H). Based on these results, 24 h post-infection was selected for subsequent mechanistic experiments.

### 3.3 PCBP2 negatively regulates the RLR-mediated antiviral signaling pathway

The RIG-I-like Receptor (RLR) signaling pathway played an important role in the antiviral immune response against RNA viruses (Yoshikawa et al., 2023, Satoh et al., 2010). RIG-I could detect RNA viruses and initiate an immune response by activating MAVS. Then, TBK1 is activated, which ultimately triggers the expression of IFN-β and the subsequent activation of ISGs including ISG12a and G1P3 (Min et al., 2020).

**Fig. 3:**
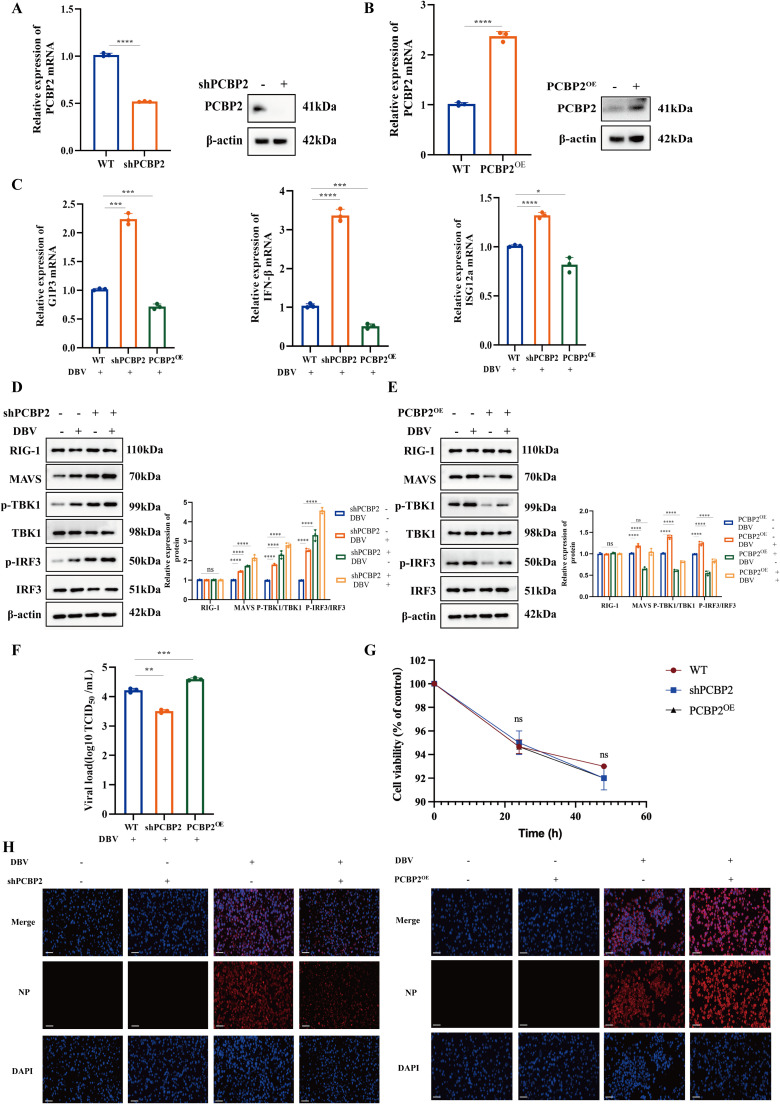
PCBP2 negatively regulates the RLR-mediated antiviral signaling pathway. (A-B) Validation of lentivirus-mediated PCBP2 knockdown (shPCBP2) and plasmid-mediated PCBP2 overexpression (PCBP2^OE^) in THP-1 cells by qRT-PCR and western blot analysis. (C) Relative mRNA expression levels of IFN-β and interferon-stimulated genes (G1P3 and ISG12a) in DBV-infected THP-1 cells with shPCBP2 or PCBP2^OE^. (D) Western blot analysis and densitometric quantification of RLR signaling components, including RIG-I, MAVS, TBK1, phosphorylated TBK1 (p-TBK1), IRF3, and phosphorylated IRF3 (p-IRF3), in DBV-infected shPCBP2 THP-1 cells. (E) Western blot analysis and densitometric quantification of RLR signaling components in DBV-infected PCBP2^OE^ THP-1 cells. (F) DBV replication levels determined by TCID_50_ assay in THP-1 cells with shPCBP2 or PCBP2^OE^ following DBV infection. (G) Cell viability of THP-1 cells with shPCBP2 or PCBP2^OE^ at 0, 24, and 48 h post-infection, measured by CCK-8 assay to confirm comparable cell proliferation among groups. (H) Immunofluorescence analysis of DBV nucleoprotein (NP) expression in shPCBP2 and PCBP2^OE^ THP-1 cells after DBV infection. Data are presented as mean ± SD from three independent experiments. *, *P* < 0.05; **, *P* < 0.01; ***, *P* < 0.001; ****, *P* < 0.0001. WT, wild-type cells.

To investigate the role of PCBP2 in regulating RLR-mediated antiviral signaling during DBV infection, THP-1 cells with lentivirus-mediated PCBP2 knockdown (shPCBP2) or plasmid-mediated PCBP2 overexpression (PCBP2^OE^) were generated and validated (Fig. 3A-B).

Quantitative RT-PCR analysis demonstrated that PCBP2 knockdown significantly enhanced DBV-induced transcription of IFN-β and interferon-stimulated genes (ISG12a and G1P3), whereas PCBP2 overexpression markedly suppressed the expression of these antiviral genes (Fig. 3C), indicating that PCBP2 functions as a negative regulator of the antiviral innate immune response.

Consistently, western blot analysis showed that, upon DBV infection, shPCBP2 cells exhibited increased total protein levels and phosphorylation of MAVS, TBK1, and IRF3, while the expression of the upstream pattern-recognition receptor RIG-I remained unchanged. In contrast, PCBP2^OE^ cells displayed reduced total protein abundance and phosphorylation of MAVS, TBK1, and IRF3, with no detectable alteration in RIG-I expression. Densitometric quantification further confirmed the opposing effects of PCBP2 knockdown and overexpression on the activation of the MAVS-TBK1-IRF3 signaling axis (Fig. 3D-E).

To determine whether PCBP2-mediated regulation of antiviral signaling impacts viral replication, DBV titers were measured by TCID_50_ assay. PCBP2 knockdown resulted in a significant reduction in viral load, whereas PCBP2 overexpression led to a marked increase in DBV replication (Fig. 3F). To exclude the possibility that the observed differences in viral replication and NP expression were caused by variations in cell number or viability, cell proliferation was assessed using the CCK-8 assay. THP-1 cells with shPCBP2 or PCBP2^OE^ exhibited comparable viability at 0, 24, and 48 h post-infection, with no significant differences among the groups (*P* > 0.05) (Fig. 3G).

Consistently, immunofluorescence analysis revealed decreased NP protein expression in shPCBP2 cells and enhanced NP expression in PCBP2^OE^ cells following DBV infection (Fig. 3H). These results indicate that the effects of PCBP2 on DBV replication were independent of cell proliferation or cytotoxicity.

Collectively, these results demonstrate that PCBP2 suppresses RLR-mediated antiviral signaling through inhibition of the MAVS–TBK1–IRF3 axis, thereby promoting DBV replication.

### 3.4 PCBP2 targets MAVS and induces its degradation during DBV infection

**Fig. 4:**
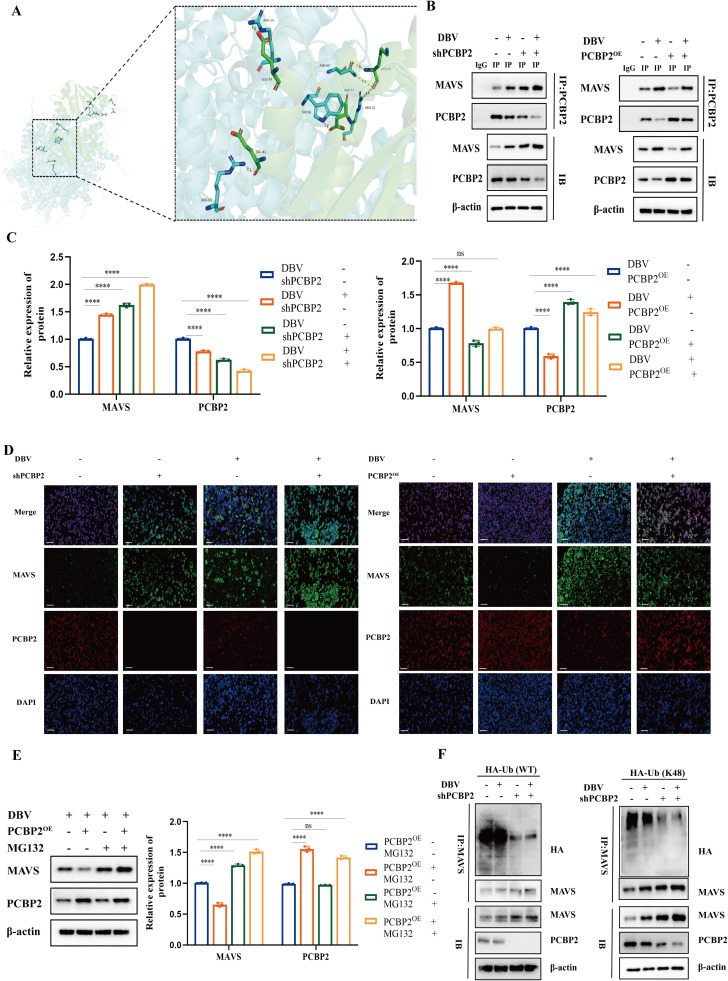
PCBP2 targets MAVS and induces its degradation during DBV infection. (A) Structural modeling using PyMOL predicted potential binding interfaces between PCBP2 and MAVS. (B) Co-immunoprecipitation (co-IP) assays showing the interaction between PCBP2 and MAVS in DBV-infected THP-1 cells under PCBP2 knockdown (shPCBP2) and overexpression (PCBP2^OE^) conditions, as detected by western blot analysis. (C) Densitometric quantification of the co-IP results shown in panel (B), normalized to the corresponding input controls. (D) Dual-immunofluorescence staining demonstrating the cytoplasmic colocalization of PCBP2 and MAVS in DBV-infected THP-1 cells. Scale bars, 20 μm. (E) THP-1 cells were infected with DBV (MOI = 1) and transfected with either an empty vector or a PCBP2 overexpression plasmid for 48 h, followed by treatment with DMSO or the proteasome inhibitor MG132 (25 μM) for the final 6 h. MAVS protein levels were analyzed by western blot. (F) Ubiquitination assays showing total ubiquitination and K48-linked ubiquitination of MAVS in DBV-infected THP-1 cells under PCBP2 overexpression or knockdown conditions. Cells were treated with MG132 (25 μM) for 6 h prior to harvest, and immunoblotting was performed with the indicated antibodies. All data are presented as the mean ± SD from three independent experiments. *, *P* < 0.05; **, *P* < 0.01; ***, *P* < 0.001; ****, *P* < 0.0001.

Based on the inhibitory role of PCBP2 in RLR-mediated antiviral signaling, we hypothesized that PCBP2 may exert its regulatory function through direct interaction with MAVS. Structural modeling using the PyMOL molecular graphics system predicted potential binding interfaces between PCBP2 and MAVS, providing a structural basis for subsequent experimental validation (Fig. 4A).

To experimentally validate this interaction, co-immunoprecipitation (co-IP) assays were performed in DBV-infected THP-1 cells under PCBP2 knockdown and overexpression conditions. Interestingly, a subtle inverse relationship was observed between the levels of precipitated PCBP2 and MAVS, which was further supported by densitometric quantification of the co-IP results (Fig. 4B-C).

In addition, dual-immunofluorescence staining revealed the cytoplasmic colocalization of PCBP2 and MAVS in DBV-infected THP-1 cells, providing spatial evidence supporting their interaction during infection (Fig. 4D). The fluorescence intensity scanning across the cellular sections (indicated by white lines) further revealed highly synchronized signal peaks for both PCBP2 and MAVS, with additional representative cells provided in Fig. S1.

We next investigated whether PCBP2-mediated downregulation of MAVS occurs via the proteasomal degradation pathway. DBV-infected cells overexpressing PCBP2 were treated with the proteasome inhibitor MG132, which markedly restored MAVS protein levels compared with untreated cells under DBV infection conditions (Fig. 4E), indicating that PCBP2-induced MAVS degradation is proteasome dependent.

Given that ubiquitination is a prerequisite for proteasome-mediated protein degradation, we further examined the ubiquitination status of MAVS. Ubiquitination assays showed that DBV infection promoted MAVS ubiquitination in PCBP2-expressing cells, whereas this effect was substantially attenuated in PCBP2-silenced cells. Notably, K48-linked ubiquitination of MAVS was significantly reduced upon PCBP2 knockdown (Fig. 4F), indicating that PCBP2 primarily facilitates MAVS degradation through K48-linked ubiquitination.

## 4 Discussion

Severe fever with thrombocytopenia syndrome (SFTS) remains a major public health threat due to its high case-fatality rate and the lack of effective vaccines or specific antiviral therapies (Casel et al., 2021). Elucidating the molecular mechanisms underlying host–virus interactions is therefore essential for understanding SFTS pathogenesis and identifying potential therapeutic targets (Yanatori and Kishi, 2019). Accumulating evidence from other viral infections has highlighted poly(rC)-binding protein 2 (PCBP2) as an important host factor involved in the regulation of innate immune responses (He et al., 2023, Wang et al., 2011). However, its role in Dabie bandavirus (DBV) infection has not been previously defined. In this study, we systematically demonstrate that PCBP2 is downregulated during DBV infection and functions as a negative regulator of antiviral innate immunity, thereby facilitating viral replication.

A key finding of our study is that PCBP2 expression is consistently reduced during DBV infection across clinical samples, animal models, and cultured cells. Single-cell transcriptomic analysis revealed a global decrease in PCBP2 mRNA expression in peripheral blood mononuclear cells (PBMCs) from SFTS patients, which was further validated by bulk qRT-PCR and western blot analyses. Importantly, lower PCBP2 expression was associated with increased disease severity and poorer clinical outcomes, suggesting that PCBP2 dysregulation is closely linked to disease progression rather than representing a nonspecific consequence of infection. These observations extend previous studies on PCBP2 in viral infections and establish its clinical relevance in SFTS.

Using an established IFNα receptor–deficient (IFNAR⁻/⁻) mouse model that is susceptible to DBV infection, we further demonstrated that PCBP2 expression is dynamically regulated in vivo. DBV infection induced a dose- and time-dependent increase in viral load accompanied by progressive weight loss, recapitulating key features of disease severity. In parallel, PCBP2 mRNA levels in peripheral blood and protein expression in target organs were markedly reduced, particularly in mice infected with a higher viral dose. Notably, because IFNAR⁻/⁻ mice lack type I interferon signaling, these results indicate that DBV-induced downregulation of PCBP2 occurs independently of canonical interferon responses, highlighting a direct or interferon-independent mechanism of PCBP2 regulation during infection.

Importantly, our data suggest that PCBP2 regulation during DBV infection is not static but instead exhibits temporal dynamics. During the early stage of infection, basal levels of PCBP2 act as a negative regulator of MAVS-mediated antiviral signaling, thereby suppressing type I interferon responses and creating a permissive environment for initial viral replication. As infection progresses and viral burden increases, host cells appear to downregulate PCBP2 expression as a compensatory response to restore antiviral signaling and limit excessive viral propagation. This dynamic regulation provides a mechanistic explanation for the apparent paradox between reduced PCBP2 expression observed in severe SFTS patients and the proviral role of PCBP2 demonstrated in vitro, reflecting different phases of host–virus interaction.

Mechanistically, our data indicate that PCBP2 suppresses RIG-I-like receptor (RLR)-mediated antiviral signaling during DBV infection. Innate immunity constitutes the first line of host defense against viral invasion (Zhang et al., 2019, Erttmann et al., 2022). Recognition of viral RNA by pattern recognition receptors (PRRs), particularly RLRs such as RIG-I, initiates downstream signaling cascades through mitochondrial antiviral-signaling protein (MAVS), leading to activation of TBK1 and IRF3 and subsequent induction of type I interferons and interferon-stimulated genes (ISGs) (Hoffmann et al., 2015, Yamada et al., 2018). In the present study, PCBP2 overexpression significantly attenuated DBV-induced activation of the MAVS–TBK1–IRF3 axis and suppressed the transcription of IFN-β and representative ISGs, whereas PCBP2 knockdown exerted the opposite effect. Notably, PCBP2 did not alter RIG-I expression, indicating that its regulatory effect occurs downstream of viral RNA sensing but upstream of TBK1 and IRF3 activation.

Further investigation revealed that PCBP2 exerts its inhibitory effect by directly targeting MAVS for proteasomal degradation. We demonstrated that PCBP2 physically interacts with MAVS and promotes its ubiquitination in DBV-infected cells. Pharmacological inhibition of the proteasome restored MAVS protein levels, confirming that PCBP2-mediated MAVS downregulation is proteasome dependent. More specifically, PCBP2 facilitated K48-linked polyubiquitination of MAVS, a canonical signal for proteasomal degradation. These findings are consistent with previous reports describing PCBP2 as a negative regulator of antiviral signaling through ubiquitin-dependent mechanisms (Wang et al., 2011), and they extend this regulatory paradigm to DBV infection.

Functionally, suppression of antiviral signaling by PCBP2 translated into enhanced viral replication. PCBP2 knockdown significantly reduced DBV titers and viral nucleoprotein expression, whereas PCBP2 overexpression promoted viral replication in THP-1 cells. These results underscore the role of PCBP2 as a host factor that favors DBV replication by dampening innate immune responses. From a broader perspective, our findings support the concept that viruses exploit host negative regulatory pathways to evade immune surveillance and establish productive infection (Horner and Gale, 2026, Wu and Chen, 2026, Yang and Zhu, 2015).

Several limitations of this study should be acknowledged. Although we incorporated both in vivo and in vitro models, the precise upstream mechanisms responsible for PCBP2 downregulation during DBV infection remain to be elucidated. In particular, it is conceivable that specific DBV structural or nonstructural proteins may directly or indirectly modulate PCBP2 transcription, stability, or post-translational modification, thereby fine-tuning host antiviral responses at different stages of infection. Additionally, although IFNAR⁻/⁻ mice provide a robust and widely accepted model for studying DBV pathogenesis, they do not fully recapitulate immune regulation in immunocompetent hosts. Future studies combining PCBP2 gain-of-function approaches with interferon-deficient and immunocompetent animal models will be valuable for fully delineating the contribution of PCBP2 to DBV pathogenesis.

In conclusion, our study identifies PCBP2 as a critical negative regulator of RLR-mediated antiviral signaling during DBV infection. By promoting K48-linked ubiquitination and proteasomal degradation of MAVS, PCBP2 suppresses type I interferon responses and facilitates viral replication. These findings provide new insights into the immune evasion strategies employed by DBV and highlight PCBP2 as a potential host-directed therapeutic target for SFTS.

## Ethics statement

The studies involving human participants were reviewed and approved by Ethics Committee of Jiangsu Provincial People's Hospital. The patients/participants provided their written informed consent to participate in this study.

## Funding

The author(s) declare that financial support was received for the research and/or publication of this article. This study was supported by the 10.13039/501100011441State Key Laboratory for Diagnosis and Treatment of Severe Zoonotic Infectious Disease (Grant No. 2024KF00004), the 10.13039/501100001809National Natural Science Foundation of China (81871242), and the Special Research Fund for Inflammatory Storm Prevention and Treatment of Major Diseases, Jiangsu Province Society of Research-Oriented Medical Sciences (Grant No. SYHKJ-XF-2025-17).

## Disclosure statement

This is a short text to acknowledge the contributions of specific colleagues, institutions, or agencies that aided the efforts of the authors.

No potential conflicts of interest were reported by the author (s).

## CRediT authorship contribution statement

**Xinyi Yu:** Writing – original draft. **Yan Dai:** Writing – review & editing. **Xuewen Ji:** Formal analysis. **Qinqin Pu:** Data curation. **Ruonan Zhang:** Software. **Mengqi Shi:** Resources. **Nannan Hu:** Validation. **Ke Jin:** Visualization. **Jin Zhu:** Supervision. **Jun Li:** Funding acquisition.

## Declaration of competing interest

This is a short text to acknowledge the contributions of specific colleagues, institutions, or agencies that aided the efforts of the authors.

No potential conflicts of interest were reported by the author (s).

## Data Availability

The original contributions presented in the study are included in the article/Supplementary material, further inquiries can be directed to the corresponding authors.

## Appendix Supplementary materials

Image, application 1

Image, application 2
