# Supplementary material for: PCBP2 inhibits antiviral innate immune responses via the MAVS-mediated signaling pathway in severe fever with thrombocytopenia syndrome
Source: Virus Res. 2026 Feb 2;365:199699. doi: 10.1016/j.virusres.2026.199699 (PMC12914862; doi:10.1016/j.virusres.2026.199699)
Supplement: Supplementary file 1 [file mmc1.docx]

**S1 Table. The primer sequences**

| **Primer** | **Sequence (5'-3')** |
| --- | --- |
| Homo-PCBP2-F | ATTATCACTTTGGCTGGACC |
| Homo-PCBP2-R | GATGGATTGTGGAATGCC |
| Homo-GAPDH-F | ACAACTTTGGTATCGTGGAAGG |
| Homo-GAPDH-R | GCCATCACGCCACAGTTTC |
| Homo-IFN-β-F | AGTAGGCGACACTGTTCGTG |
| Homo-IFN-β-R | AGCCTCCCATTCAATTGCCA |
| Homo-ISG12a-F | TGCCATGGGCTTCACTGCGG |
| Homo-ISG12a-R | CTGCCCGAGGCAACTCCACC |
| Homo-G1P3-F | AGGCTCCGGGCTGAAGATT |
| Homo-G1P3-R | TGCAAGTGAAGAGCAGCAGGT |
