# Supplementary figures and images for: PCBP2 inhibits antiviral innate immune responses via the MAVS-mediated signaling pathway in severe fever with thrombocytopenia syndrome
Source: Virus Res. 2026 Feb 2;365:199699. doi: 10.1016/j.virusres.2026.199699 (PMC12914862; doi:10.1016/j.virusres.2026.199699)

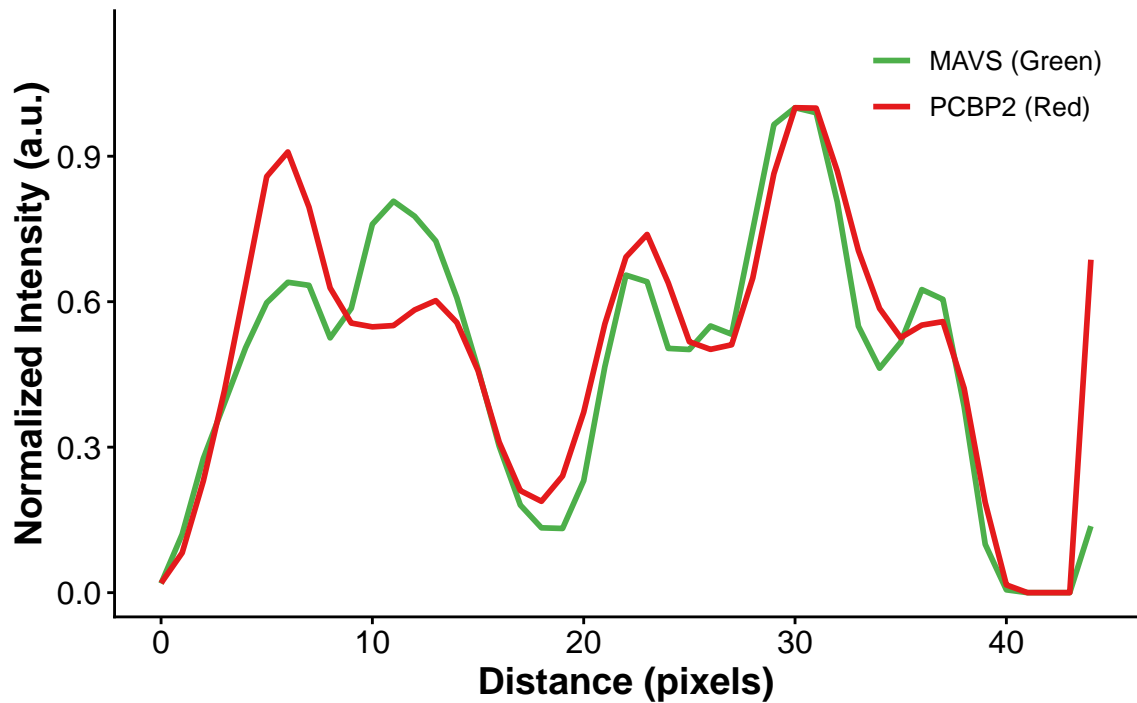

Supplement: Supplementary file 2 [file mmc2.pdf]
